# Interaction of Antimicrobial Lipopeptides with Bacterial Lipid Bilayers

**DOI:** 10.1007/s00232-019-00068-3

**Published:** 2019-05-16

**Authors:** Ganesh Shahane, Wei Ding, Michail Palaiokostas, Helena S. Azevedo, Mario Orsi

**Affiliations:** 1 Institute of Bioengineering, Queen Mary University of London, Mile End Road, London, E1 4NS UK; 2 School of Engineering & Materials Science, Queen Mary University of London, Mile End Road, London, E1 4NS UK; 3 Department of Applied Sciences, University of the West of England, Coldharbour Lane, Bristol, BS16 1QY UK

**Keywords:** Antimicrobial lipopeptides, Lipid bilayers, Molecular simulation, Molecular dynamics

## Abstract

The resistance of pathogens to traditional antibiotics is currently a global issue of enormous concern. As the discovery and development of new antibiotics become increasingly challenging, synthetic antimicrobial lipopeptides (AMLPs) are now receiving renewed attention as a new class of antimicrobial agents. In contrast to traditional antibiotics, AMLPs act by physically disrupting the cell membrane (rather than targeting specific proteins), thus reducing the risk of inducing bacterial resistance. In this study, we use microsecond-timescale atomistic molecular dynamics simulations to quantify the interaction of a short AMLP (C16-KKK) with model bacterial lipid bilayers. In particular, we investigate how fundamental transmembrane properties change in relation to a range of lipopeptide concentrations. A number of structural, mechanical, and dynamical features are found to be significantly altered in a non-linear fashion. At 10 mol% concentration, lipopeptides have a condensing effect on bacterial bilayers, characterized by a decrease in the area per lipid and an increase in the bilayer order. Higher AMLP concentrations of 25 and 40 mol% destabilize the membrane by disrupting the bilayer core structure, inducing membrane thinning and water leakage. Important transmembrane properties such as the lateral pressure and dipole potential profiles are also affected. Potential implications on membrane function and associated proteins are discussed.

**Electronic supplementary material:**

The online version of this article (10.1007/s00232-019-00068-3) contains supplementary material, which is available to authorized users.

## Introduction

The emergence and spread of bacterial strains resistant to antibiotics represent a formidable global health problem. Alternative compounds such as antimicrobial peptides (AMPs) have therefore been developed to have broad-spectrum activity while being less likely to trigger bacterial resistance (Koczulla and Bals 2003). Most AMPs are cationic in nature due to the large number of positively charged lysine and arginine residues, thus promoting attractive electrostatic interactions with anionic bacterial membranes (while membranes of mammalian cells are approximately neutral). AMPs are known to destabilize bilayers either by toroidal pore formation (Sengupta et al. 2008) or by a number of proposed models such as the carpet model (Shai and Oren (2001) or the barrel-stave model (Ehrenstein and Lecar 1977), leading to outflow of crucial nutrients and ions and eventually bacterial cell death. However, AMPs are not without drawbacks, and despite still holding promise, they have been shown to be susceptible to breakdown by peptidases when used in vivo (Hancock and Sahl 2006; Straus and Hancock 2006). Moreover, AMPs are far larger than usual drug-like molecules and thus more complex and expensive to synthesize.

**Table 1:** Simulation systems

System	$\hbox {N}_{\mathrm{lipids}}$	$\hbox {N}_{\mathrm{lipopeptides}}$	$\hbox {N}_{\mathrm{water}}$	$\hbox {N}_{\mathrm{ions}}$
Bacterial	PE (88), PG (44)	0	5940	44 (+)
10% AMLP	PE (88), PG (44)	14	6141	2 (+)
25% AMLP	PE (100), PG (50)	50	7520	100 (−)
40% AMLP	PE (100), PG (50)	100	11850	250 (−)

$\hbox {N}_{\mathrm{lipids}}$ is the total number of lipids, $\hbox {N}_{\mathrm{lipopeptides}}$ is the total number of C16-KKK lipopeptides, $\hbox {N}_{\mathrm{water}}$ is the total number of water molecules, and $\hbox {N}_{\mathrm{ions}}$ is the total number of ions in the system. Lipid names are abbreviated as PE (POPE) and PG (POPG)

**Table 2:** Lateral diffusion coefficients (10^−7^ cm^2^/s)

System	POPE	POPG
Bacterial	5.42 ± 0.25	5.01 ± 0.25
10% AMLP	0.77 ± 0.06	0.85 ± 0.08
25% AMLP	0.71 ± 0.04	0.75 ± 0.06
40% AMLP	1.22 ± 0.06	1.32 ± 0.08

**Table 3:** Elastic properties for all bilayer systems

System	$\kappa ^{m}$ ($10^{-20}\, \hbox {J}$)	$\text {c}^m_0$ ($\hbox {nm}^{-1}$)
Bacterial	4.48 ± 0.5	0.009 ± 0.005
10% AMLP	4.97 ± 0.4	− 0.014 ± 0.006
25% AMLP	4.32 ± 0.9	0.002 ± 0.001
40% AMLP	1.55 ± 0.1	0.016 ± 0.007

**Table 4:** Summary of conclusions

System	Lipids	$A_{\mathrm{L}}$	$d_{\mathrm{HH}}$	$\rho (z)$	$S_{\mathrm{CD}}$	$P_{\mathrm{w}}$	$D_{\mathrm{L}}$	$\varPi (z)$	$\kappa ^{m}$	$\text {c}^m_0$	$\varPsi (z)$
Bacterial	PE / PG	= / =	=	=	=	=	= / =	=	=	flat	=
10% AMLP	PE / PG	$\downarrow$ / $\downarrow$	=	=	$\uparrow$ / $\uparrow$	$\downarrow$	$\downarrow$ / $\downarrow$	$\downarrow$ (heads)	=	=	$\uparrow$
25% AMLP	PE / PG	$\downarrow$ / $\downarrow$	$\downarrow$	$\uparrow$ (tails)	$\downarrow$ / $\downarrow$	$\uparrow$	$\downarrow$ / $\downarrow$	$\downarrow$	=	=	$\uparrow$
40% AMLP	PE / PG	$\uparrow$ / $\downarrow$	$\downarrow$	$\uparrow$ (tails)	$\downarrow$ / $\downarrow$	$\uparrow$	$\downarrow$ / $\downarrow$	$\downarrow$	$\downarrow$	=	$\downarrow$ (tails)

Conclusions derived in this study are summarized as above, wherein properties of the pure bacterial system are compared with systems containing AMLPs. Upward ($\uparrow$) and downward ($\downarrow$) arrows, respectively, correspond to increases and decreases in magnitudes of the said properties in comparison to those of the bacterial bilayer. Properties with two arrows (e.g., $\downarrow$ / $\downarrow$) that are separated by a “/” correspond to an increase or a decrease in magnitudes of properties of PE / PG lipids in the said system in comparison to those of the bacterial bilayer. $P_{\mathrm{w}}$ is the water permeability coefficient as reported in Fig. 5b

**Fig. 1:**
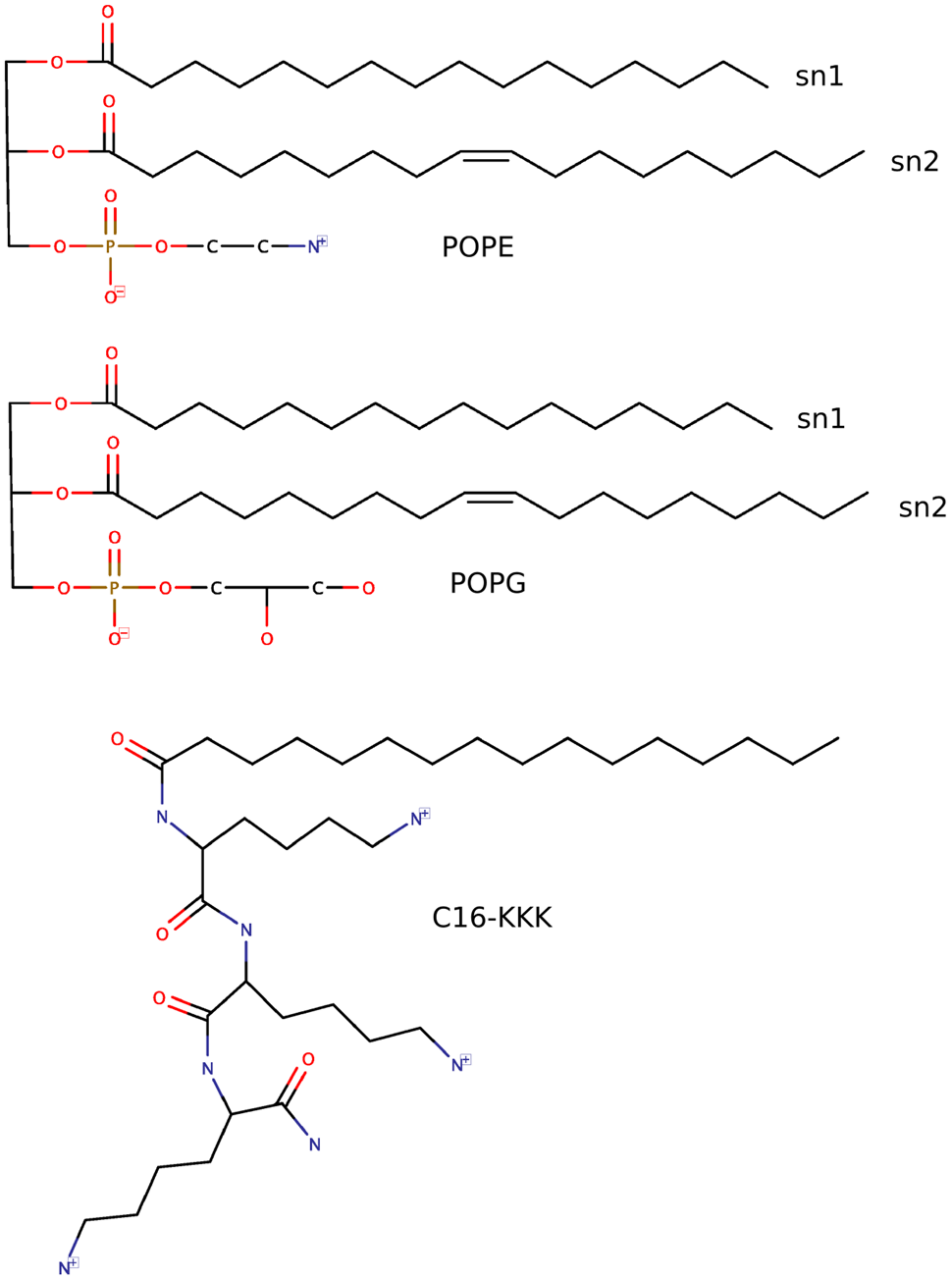
Molecular structures of POPE (top), POPG (middle), and C16-KKK (below). Hydrogens are not shown for clarity

**Fig. 2:**
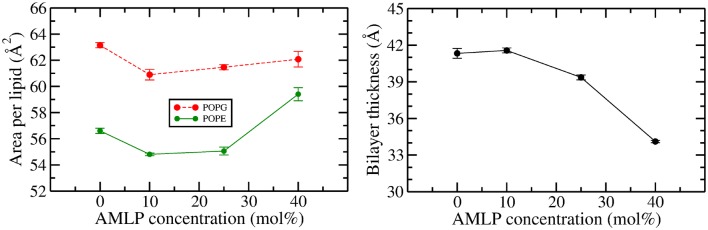
Area per lipid (left) and bilayer thickness (right) values as a function of PA concentration. The area per lipid values were calculated for POPE and POPG lipids individually. Error bars represent standard errors

**Fig. 3:**
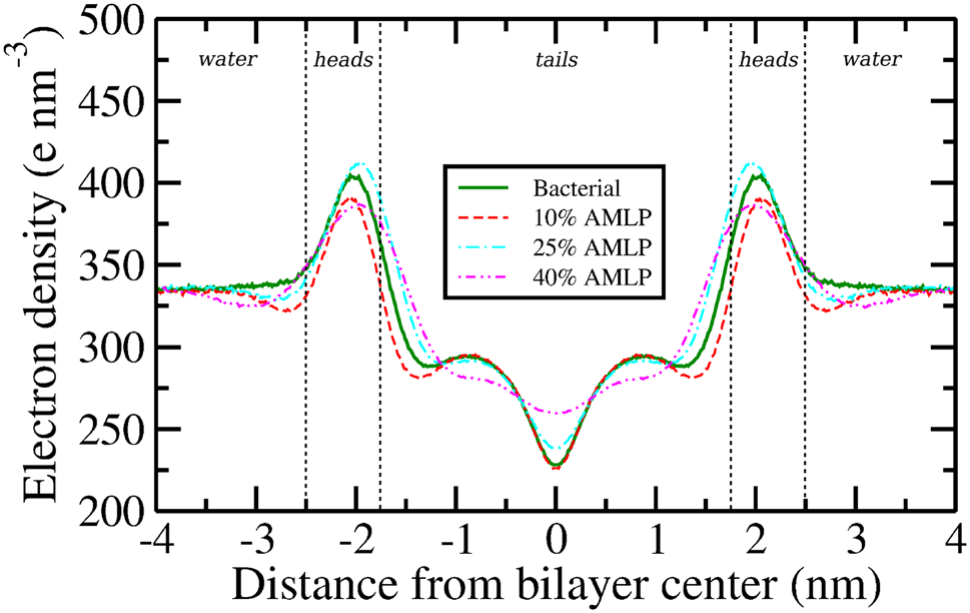
Electron density profiles. Error bars, omitted here for clarity, are reported in the supplementary material

**Fig. 4:**
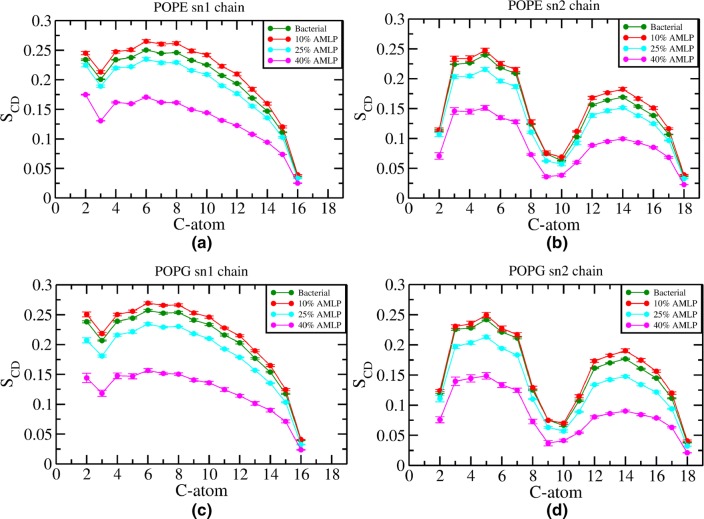
Deuterium-order parameters ($\hbox {S}_{\mathrm{CD}}$) for **a** POPE sn-1 chain, **b** POPE sn-2 chain, **c** POPG sn-1 chain, and **d** POPG sn-2 chain. Dashes represent error bars

**Fig. 5:**
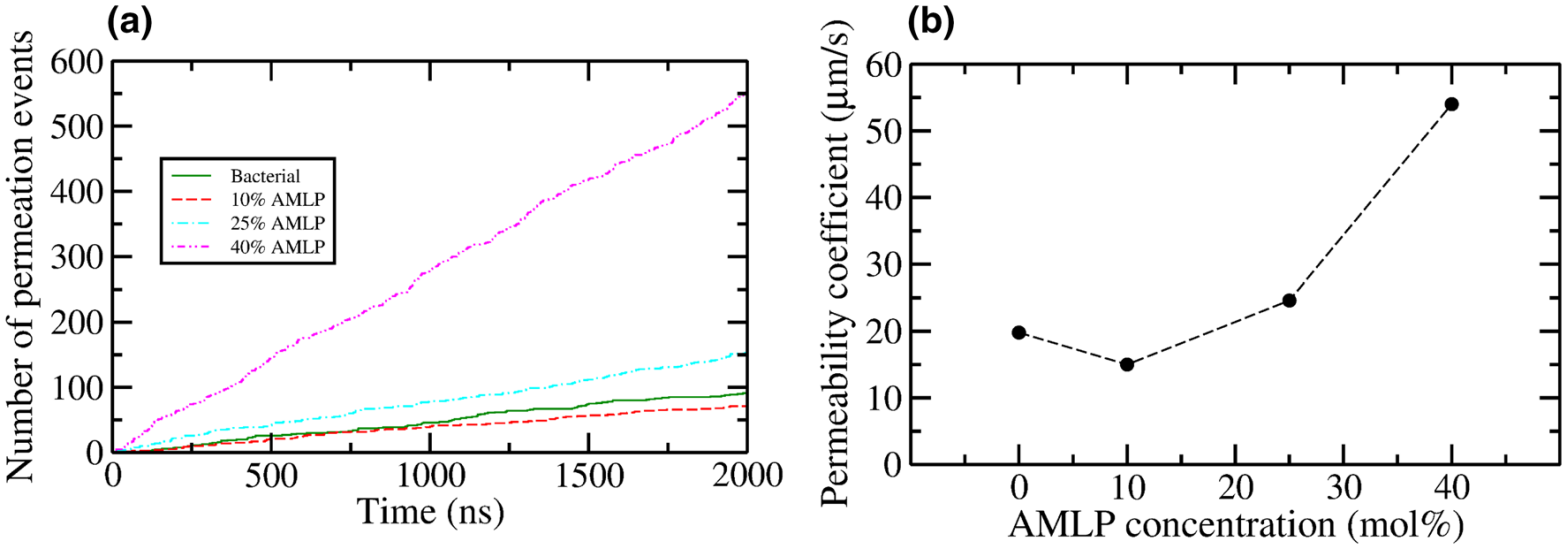
Measurement of water permeation. **a** Cumulative number of water permeation events as a function of simulation time; **b** water permeability coefficient as a function of AMLP concentration

To develop drugs that retain antimicrobial properties and circumvent drawbacks, Avrahami and Shai developed an approach to conjugate fatty acids to short non-active cationic peptides, providing the resulting molecules with antimicrobial properties while controlling their size (Avrahami and Shai 2004; 2003). Specifically, in pioneering studies by Shai and coworkers (Makovitzki et al. 2006; 2008), a set of di-, tri-, and tetrapeptides were attached to palmitoyl chains through their N-terminus, creating a new class of short cationic lipopeptides. Several of them revealed strong antibacterial and antifungal activities as monitored in vitro, without causing any significant hemolysis. Experiments were performed to measure macroscopic properties such as dye leakage from calcein-encapsulated small unilamellar vesicles (SUVs) via membrane rupture, measuring entrance of dyes and probes in ruptured cells, determination of cell growth inhibition from microplate autoreaders, and visualization of cell damage using transmission electron microscopy (TEM). Another subsequent study revealed the effectiveness of such lipopeptides for clearing infections in vivo (Vallon-Eberhard et al. 2008). The structural simplicity of these antimicrobial lipopeptides (AMLPs) makes them relatively easy to synthesize and analyze in relation to their antimicrobial mechanisms.

**Fig. 6:**
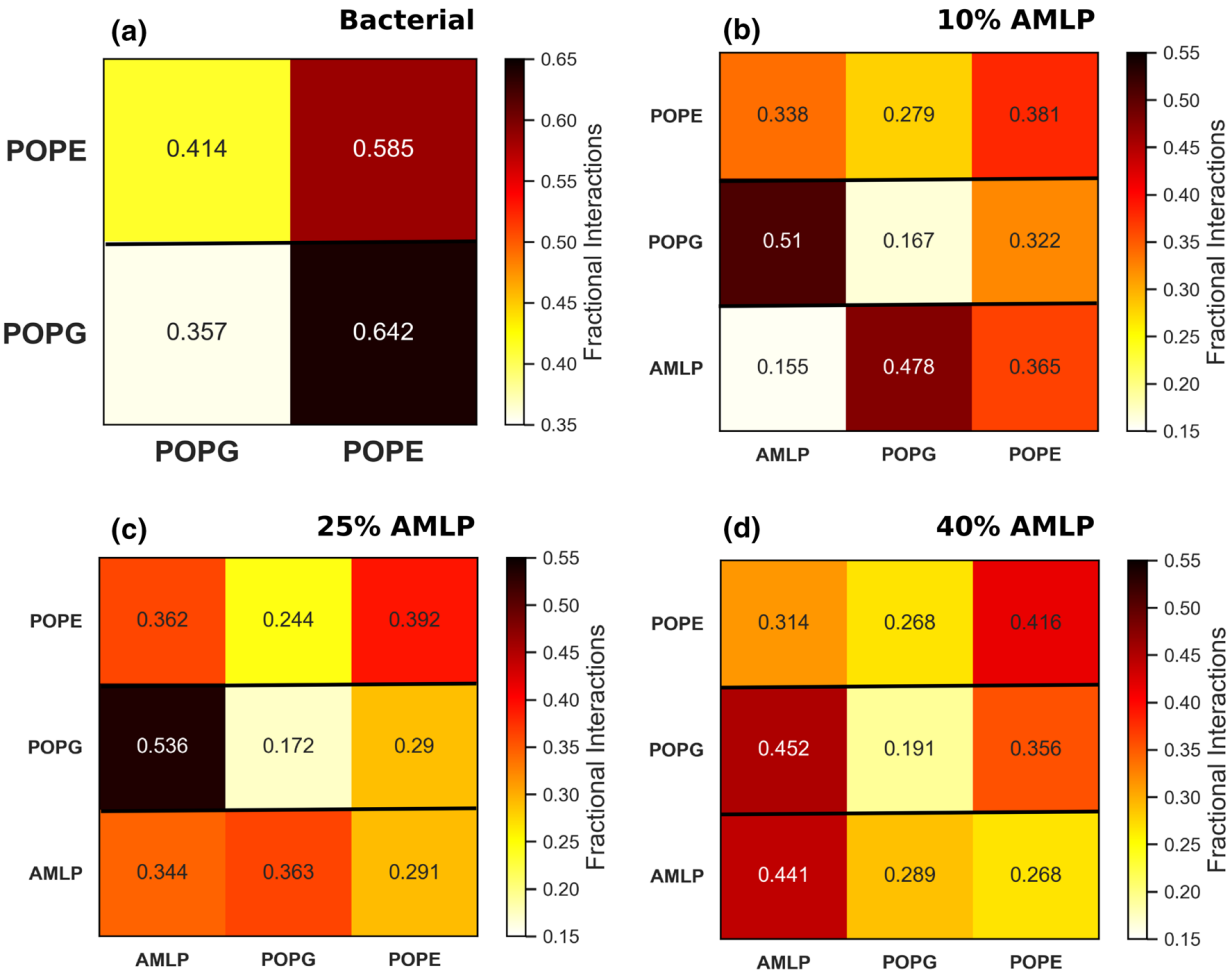
Fractional interaction matrices for all the simulated bilayers. Each fractional interaction is the relative number of contacts between lipids as compared to all other contacts. Two lipids are defined as being in contact if the distance between their phosphate atoms is less than 6 Å. For AMLPs, the oxygen atom of the peptide bond connecting the first and the second lysine is considered for calculations. For the bacterial bilayer, a fully random distribution of the two lipids would result in a fraction of 0.5, while for the systems with AMLPs the fraction would be 0.33. The fractions are arranged such that in each row the sum of all fractions for each component would be equal to 1. This figure shows fractional interaction matrices of **a** bacterial bilayer, **b** 10% AMLP, **c** 25% AMLP, and **d** 40% AMLP

**Fig. 7:**
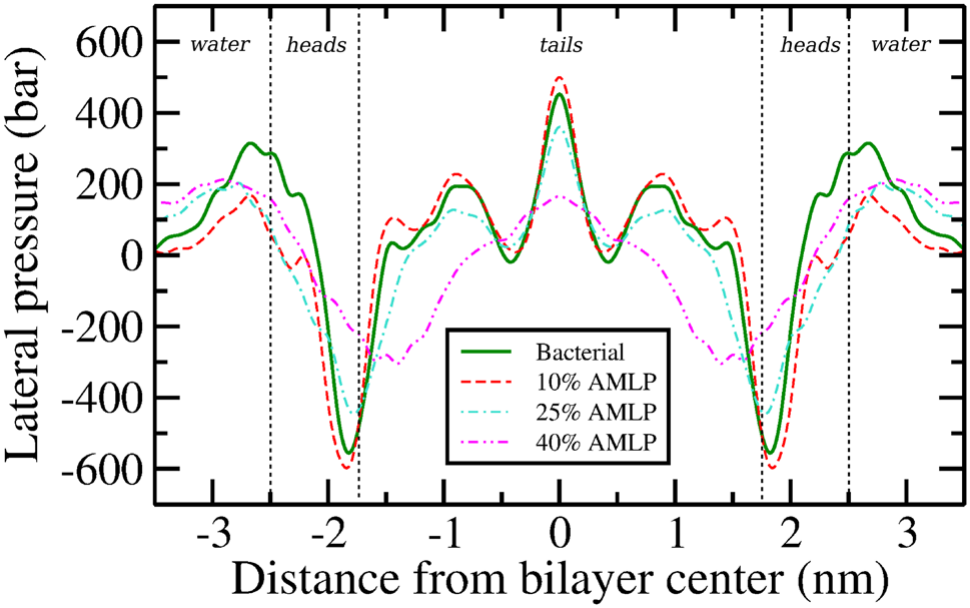
Lateral pressure profiles. Error bars, omitted here for clarity, are reported in the supplementary material

**Fig. 8:**
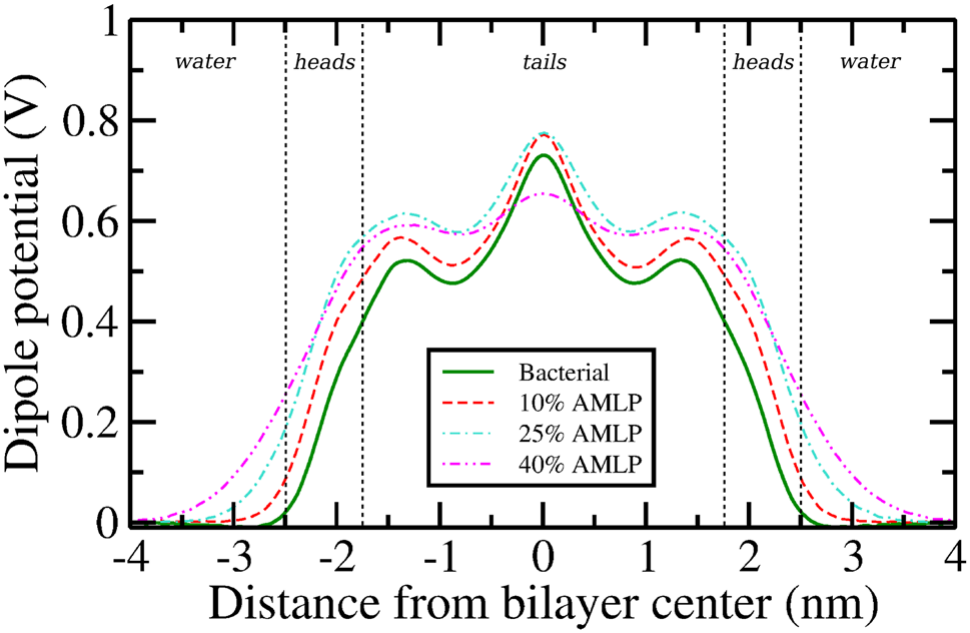
Dipole potential profiles. Error bars, omitted here for clarity, are reported in the supplementary material

Though AMLPs have antibacterial and antifungal features, their mode of action has been revealed to be notably different from that of AMPs (Makovitzki et al. 2006, 2008; Horn et al. 2013; Sikorska et al. (2014; Grasso et al. 2018). A number of molecular dynamics (MD) simulation studies have shown that interactions with bacterial lipid bilayers generally follow a three-step process: initial self-assembly of AMLPs into micelles, binding and insertion of the micelle into the lipid bilayer, and subsequent scattering of the AMLPs throughout the bilayer (Horn et al. 2013; Sikorska et al. 2014). Particularly, the self-assembly and cluster formation processes with peptide sequences of varying lengths were meticulously characterized using coarse-grained (CG) models by Sikorska et al. (2014); a key finding from the study was that an increase in peptide sequence increases its critical micellar concentration (CMC) and decreases the aggregation number. While increased steric interactions in the AMLP headgroups were found to be responsible for an increase in CMC, the reason for a decrease in aggregation number was intuitively ascribed to increased electrostatic repulsion between micelles preventing their fusion into larger ones. Though AMLPs are independently capable of spontaneous self-assembly, the studies also show that the process can occur concurrently in association with the bilayer surface (Sikorska et al. 2014). Binding of AMLP clusters is then accompanied by formation of 1-palmitoyl-2-oleoyl-sn-glycero-3-phosphoglycerol (POPG)-rich domains at the binding site, caused by preferential electrostatic interactions of negatively charged PG headgroups with cationic lysines (K) (Horn et al. 2013; Sikorska et al. 2014).

While it is clear that considerable work has been done with respect to analyzing self-assembly and binding of AMLPs to model bacterial bilayers, previous studies (as briefly reviewed above) typically lack systematic analyses of the effects of AMLPs concentration on key structural, dynamical, transmembrane, and elastic properties. To address this issue, in this study we use atomistic MD simulations to examine and quantify crucial properties of a simple model of gram-negative bacterial inner membrane bilayer for a range of AMLP concentrations. The bacterial bilayer composition is modeled as a mixture of POPE (1-palmitoyl-2-oleoyl-sn-glycero-3-phosphoethanolamine) and POPG (1-palmitoyl-2-oleoyl-sn-glycero-3-phosphoglycerol) lipids in a 2:1 ratio, as in previous work (Horn et al. 2012, 2013). The chosen AMLP consists of a 16-carbon aliphatic chain bound to three lysine residues at the N-terminus (thus designated as C16-KKK) and previous experimental research highlighted its potent antibacterial and antifungal activities (Makovitzki et al. 2008). By studying bilayers incorporating different lipopeptide concentrations, we aim at systematically quantifying the changes induced on key membrane physical properties. Specifically, we simulate a pure bacterial bilayer system, as well as three other systems comprising AMLPs at increasing concentrations of 10, 25, and 40 mol%. Fundamental structural properties such as area per lipid, bilayer thickness, electron density profiles, and deuterium-order parameters are investigated. Water permeation events and permeability coefficients are subsequently quantified over the microseconds-long trajectories. Furthermore, we calculate transmembrane lateral pressure and dipole potential profiles, two fundamental and yet often overlooked properties that play crucial roles in many membrane processes (Shearman et al. 2006; Wang 2012; Mouritsen 2005; Palaiokostas et al. 2018). The lateral pressure profile characterizes the inhomogeneous distribution of lateral stresses as a function of depth inside the bilayer. Given the nanoscopic thickness of lipid membranes, experimental determination of internal stresses is extremely difficult. Theory and simulation data predict the lateral pressure to be highly sensitive to depth, with magnitudes varying over several hundred bars across the membrane thickness (Cevc and Marsh 1987; Samuli and Vattulainen 2010). Significant forces therefore act on embedded proteins and permeating molecules (van den Brink-van der Laan 2004; Marsh 2007; Samuli and Vattulainen 2010). Moreover, the lateral pressure profile also underlies important elastic parameters, such as the bending rigidity modulus and spontaneous curvature, which determine the bilayer ability to bend and deform. As a consequence, the pressure profile is central to many membrane-related biological processes (Liu et al. 2006; McMahon and Gallop 2005; Chernomordik et al. 1995; Sergey and Bezrukova 2000; Cafiso 1998; Attard et al. 2000; Botelho et al. 2006; Orsi et al. 2011). The dipole (or electrostatic) potential arises from the preferential alignment of the dipole moments of water and lipid molecules; similar to the pressure profile, the dipole potential is also a transmembrane property of paramount biological relevance that is extremely difficult to study experimentally (Dreyer et al. 2013; Wang 2012). In this work, we quantify the effects of different concentrations of C16-KKK AMLP on the aforementioned properties of model bacterial bilayers, and discuss implications for antimicrobial activity and membrane function.

## Methods

### System Preparation

The main details of the lipid–lipopeptide bilayer systems under investigation are reported in Table 1. Four different bacterial bilayers with a 2:1 PE:PG lipid ratio were generated using Membrane Builder (Wu et al. 2014; Jo et al. 2009; 2007) from CHARMM-GUI (Jo et al. 2008). Of these, three systems were modified to incorporate the C16-KKK AMLPs (molecular structures are reported in Fig. 1). The AMLPs were created using  Marvinsketch (2016) and were inserted throughout both bilayer leaflets by first concatenating the relevant coordinate files and then manually adjusting the AMLPs positions using the ‘move’ $\Rightarrow$ ‘molecule’ functions in VMD (Humphrey et al. 1996), thus producing three different bacterial bilayer systems with lipopeptide concentrations of 10, 25, and 40 mol%. The AMLPs locations were chosen to be approximately equidistant from one another within each leaflet. A minimization was conducted to remove any steric clashes between the lipids and lipopeptides using the steepest descent algorithm (Abraham et al. 2018). The bilayers were then solvated with the TIP3P water model as implemented in CHARMM36 (MacKerell et al. 1998; Klauda et al. 2010), followed by the addition of counterions to neutralize the overall charge. The systems were subjected to a final minimization step to ensure removal of any close contacts.

### Simulation Protocol

All-atom simulations were performed using GROMACS version 5.1 (Van Der Spoel et al. 2005; Hess et al. 2008) and CHARMM36 force field (MacKerell et al. 1998; Klauda et al. 2010), under semi-isotropic NPT conditions (1 atm and 310 K). Each system was equilibrated with the Berendsen barostat (Berendsen et al. 1984) for 10 ns, and was then simulated for further 2 $\mu$s using the Parrinello–Rahman barostat (Parrinello and Rahman 1981) with a coupling time constant of 2 ps and (default) compressibility of $4.5\cdot 10^{-5}$ bar^-1^. The velocity rescale thermostat (Bussi et al. 2007) was used with a coupling time constant of 0.1 ps. A time step of 2 fs was used for all simulations. The van der Waals and coulombic interactions were shifted to zero between 1 and 1.2 nm and 0–1.2 nm, respectively. The Particle Mesh Ewald (PME) algorithm was used for computing long-range electrostatic interactions. A grid-based search procedure was used to update neighbor list every five steps. The SETTLE algorithm (Shuichi and Kollman 1992) was used to constrain bonds and angles in water molecules. All other hydrogen-related bonds were constrained using the LINCS algorithm (Berk et al. 1997), with two iterations in every step for correcting rotational effects and a numerical expansion up to fourth order. Conventional periodic boundary conditions were applied in all three dimensions.

### Data Analysis

We evaluated the following properties: area per lipid ($A_{\mathrm{L}}$), bilayer thickness ($d_{\mathrm{HH}}$), electron density profile ($\rho (z)$), fractional interactions, deuterium-order parameters ($S_{\mathrm{CD}}$), water permeation, lateral diffusion coefficient ($D_{\mathrm{L}}$), lateral pressure profile ($\varPi (z)$), dipole potential profile ($\varPsi (z)$), bending modulus ($\kappa ^{m}$), and spontaneous curvature ($\text {c}^m_0$). Data analysis was performed over the last 251–2000 ns of each production run, while the first 250 ns were regarded as equilibration. Properties of interest were sampled every 250 ps, except for water permeation whereby a frequency of 50 ps was instead used to accurately record permeation events. Averages and standard errors were estimated using seven trajectory blocks of 250 ns length each. Transmembrane properties were averaged over the two monolayers and symmetrized with respect to the bilayer center.

To estimate the average area per lipid $A_{\mathrm{L}}$, we used the freely available tool APL@VORO (Lukat et al. 2013). The phosphate atom in the lipid headgroup was used as ’key atom’ to project coordinates on a plane and evaluate the relevant Voronoi diagrams. $A_{\mathrm{L}}$ values for POPE and POPG lipids were calculated separately in both monolayers and averaged to better determine the effects lipopeptides have on individual lipids. The electron density profiles were computed using the *gmx density* tool in GROMACS, with the simulation regions divided into 500 slabs along the *XY*-plane. The bilayer thickness $d_{\mathrm{HH}}$ was computed as the head-to-head (P-P) distance between two peaks of the lipid phosphate electron density profiles. Order parameters were obtained using the $calc\_op.tcl$ script which produces correct order parameters especially for the unsaturated lipid tails as demonstrated in a previous study (Piggot et al. 2017). Water permeation events were calculated using *gmx select* wherein the number of water oxygens with z-coordinate within the defined boundaries of the bilayer core is counted in every frame.

Normalized fractional interactions were computed for the three components POPE, POPG, and C16-KKK AMLP using a procedure previously employed to characterize the degree of lipid associations in complex membranes (Koldsø et al. 2014) and phase formations (de Jong et al. 2013). In particular, the relative number of contacts of a component was calculated with respect to each of the other components. The total number of contacts within a distance of 6 Å  for each component was obtained using the *gmx mindist* tool in GROMACS and then corrected for the total number of molecules in the respective systems. The fractional interaction was then obtained as the ratio of number of contacts with a particular component to the total number of contacts formed with all components. For a two- and three-component system, a fraction of 0.5 and 0.33, respectively, correlate to a randomly mixed bilayer. Such interactions are not necessarily symmetric since the clustering and density of specific components will, for example, make the number of contacts between lipid X and lipid Y different from lipid Y contacts with lipid X as a consequence of number of nearest neighbors.

Lipid lateral diffusion coefficients were obtained from the linear-fitted slope of averaged two-dimensional mean square displacement (MSD) from *gmx msd*, with the initial reference point reset every 50 ns. For the lateral pressure profile, a custom version of GROMACS, the GROMACS-LS package (Vanegas et al. 2014; Torres-Sánchez et al. 2015) was used to rerun trajectories and output local stress tensors in 3D. The long-range electrostatic solver is not utilized in GROMACS-LS and hence an increased cut-off distance of 2 nm was used for Coulomb interactions as suggested by the package developers (Vanegas et al. 2014). The transmembrane dipole potential was calculated using the *gmx potential* tool in GROMACS.

## Results

### Structural Properties

Calculated area per lipid ($A_{\mathrm{L}}$) and bilayer thickness ($d_{\mathrm{HH}}$) values for all the systems are shown in Fig. 2, with the numerical values reported in the supplementary information (Table S1). For the AMLP-free bacterial bilayer, both lipids exhibit $A_{\mathrm{L}}$ values in agreement with previous literature results from experimental (Rand et al. 1988; Kučerka et al. 2012) and simulation (Skjevik et al. 2015; Tolokh et al. 2009) studies. As shown in Fig. 2(left), both lipids display a similar trend in the presence of AMLPs. Specifically, the area per lipid $A_{\mathrm{L}}$ decreases upon addition of 10% AMLPs. However, it should be noted that the quantitative decrease is quite small, of ~ 3%. A gradual increase in AMLP concentration to 25 and 40% results in a corresponding increase in $A_{\mathrm{L}}$. Interestingly, by comparing the extreme cases of pure bacterial bilayer and 40% AMLPs, for POPG lipids there is only a slight net reduction of $\sim\,$1.7% while for POPE lipids we can observe a net increment of $\sim\,$5% in $A_{\mathrm{L}}$ values (with respect to the pure, AMLP-free system).

The data for bilayer thickness $d_{\mathrm{HH}}$ are displayed in Fig. 2(right). It can be noted that $d_{\mathrm{HH}}$ does not change significantly upon the addition of 10% AMLP. However, further additions of lipopeptides at 25 and 40% concentration bring about significant decreases in $d_{\mathrm{HH}}$. In particular, the $d_{\mathrm{HH}}$ decrease reaches $\sim\,$17% for the difference between the pure and the 40% AMLP systems. This can be seen clearly in the electron densities calculated for the lipid phosphate atoms, reported in Fig. S2 of the supplementary material.

The electron density profiles ($\rho (z)$) for all simulated bilayers are shown in Fig. 3. Overall, the addition of lipopeptides is accompanied by variable changes in peak magnitudes and widths. The most significant effects are observed for the 40% AMLP concentration, whereby the profile becomes considerably smoother compared to the pure bilayer system, due to a decrease in the magnitude of the main peaks and central trough, as well as an increase in corresponding width. In particular, in the tails region, this effect can be interpreted as an increase in disorder brought about by the high concentration of AMLPs. The increase in electron densities in the bilayer tails core for the 25 and 40% AMLP systems also indicates lipid chain interdigitation, which we further confirmed by visualization in VMD (Humphrey et al. 1996) (see simulation snapshots in Fig. S3 of the supplementary material). Interdigitation of lipid tails is also consistent with the observed reduction in membrane thickness.

### Order Parameters

The acyl chain deuterium-order parameters ($S_{\mathrm{CD}}$) were calculated to quantify the effect of changes in the lipopeptide concentration on the structure of the bilayer hydrophobic core:

1 $$\begin{aligned} S_{\mathrm{CD}} = \frac{1}{2} \langle 3 {\text{cos}}^2 \theta - 1 \rangle ,\end{aligned}$$

where $\theta$ is the angle between the C-D vector and the axis normal to the bilayer surface. In Fig. 4, we show the average order parameters for the acyl chain carbon atoms of PE and PG lipids in all simulated systems. The $S_{\mathrm{CD}}$ values for all *sn*-1 chains (Fig. 4a, c) exhibit similar qualitative characteristics, in that they decrease from the position of the glycerol carbon to the end of the chain, a trend usually seen in fully saturated hydrocarbon tails. The *sn*-2 chains (Fig. 4b, d) also all share similar qualitative features, with the typical trough midway along the chains corresponding to the double-bonded carbon atoms.

The presence of lipopeptides at 10% concentration marginally increases the overall order of the PE and PG lipid tails, an observation consistent with the study of a similar lipopeptide (C16-KGGK) done by Horn et al. (2013). However, at higher lipopeptide concentrations significant drops in order parameters are observed, especially for the 40% system. This sharp increase in tails disorder is expected to promote water leakage, which suggests a possible antimicrobial mode of action; this hypothesis is further investigated in the following section. Moreover, in typical (peptide free) bilayers it is common to observe an anticorrelation between lipid-order parameters and lipid area, i.e., lower areas usually correspond to higher-order parameters. This relation is disrupted by the presence of AMLPs in our simulations, as it can be seen by comparing lipid areas (Fig. 2) with order parameters (Fig. 4).

### Water Permeation

To measure any water leakage effect induced by the observed change in order parameters, we investigated the permeation of water molecules into the hydrophobic region of all simulated membranes. We defined a region in the bilayer core comprising the volume within 3 Å  from the $z=0$ plane at the bilayer center (see also Fig. S4 in the supporting information). As in previous work (Hong et al. 2014), a permeation event is recorded when a water molecule enters this region. It should be noted that while this approach captures all permeation events across the membrane, it may also produce false positives, whereby a counted water molecule fails to fully penetrate across and instead bounces back into the water phase where it came from. Though this limitation will prevent direct comparisons with standard experimental permeation measurements, we are still able to compare permeation counts in relation to different AMLP concentrations.

Figure 5a shows the cumulative number of permeation events during the simulations conducted. In particular, we recorded a total of 91, 71, 154, and 554 permeation events at the end of 2000 ns trajectory run in bacterial, 10, 25, and 40% AMLP systems, respectively, corresponding to, on average, a permeation event every $\sim\,$22, $\sim\,$28, $\sim\,$13, and $\sim\,$3.6 ns, respectively. Compared to the bacterial bilayer, water permeation marginally decreases for the 10% AMLP system, increases considerably for the 25% AMLP system, and increases manifold for the 40% AMLP system. We then calculated corresponding permeability coefficients $P_{\mathrm{W}}$ using a previously reported approach (Marrink et al. 2004; Orsi et al. 2008, 2010) based on Fick’s law:

2 $$\begin{aligned} P_{\mathrm{W}} = \frac{J_{\mathrm{W}}}{\Delta C_{\mathrm{W}} A} \end{aligned},$$

where $J_{\mathrm{W}}$ is the unidirectional flux of water (taken as half the total bidirectional permeation count) per total simulation time, *A* is the interfacial area, and $\Delta C_{\mathrm{W}}$ is the water concentration gradient calculated as $\Delta C_{\mathrm{W}} = \text {C}^{water\,phase}_W - \text {C}^{hydrocarbon\,core}_W = 30\, \hbox {nm}^{-3}$, assuming $\text {C}^{hydrocarbon\,core}_W = 0 \hbox {nm}^{-3}$. The permeability coefficients as a function of AMLP concentration are depicted in Fig. 5b (numerical values can be found in the supplementary information Table S1). It can be seen that water permeation is considerably enhanced for the 40% AMLP system. Such an effect is correlated with the previously observed decrease in order parameters and increase in lipid area at 40% AMLP concentration.

### Fractional Interactions

To better understand the nature of lipid–lipid and lipid–AMLP interactions, we calculated fractional interactions between them in all the simulated systems; results are displayed in Fig. 6. In the bacterial bilayer, PE lipids interact favorably with both PG and other PE lipids with $\sim\,$41 and $\sim\,$59% of total contacts, respectively (Fig. 6a). PG lipids however interact almost only with PE ($\sim\,$64% of all contacts) and rarely with other PG lipids.

The preference of PG–PE interactions in bacterial bilayers (Murzyn et al. 2005) and the ‘stabilizing effect’ PG lipids have on PE lipids (Tari and Huang 1989) are considered as the main regulatory mechanisms evolved by bacteria to control membrane permeability. Such behavior of PG lipids could be intuitively expected due to the presence of a negative charge on its headgroup, causing increased electrostatic repulsion between neighboring PG lipids and more favorable interactions with PE lipids via hydrogen bonding. These observations are in reasonable agreement with a previous MD study on the interaction between PE and PG lipids in a model bacterial bilayer by Murzyn et al. (2005). Introducing AMLPs in bacterial bilayers however disrupts this equilibrium of PG–PE interactions. PG lipids across all concentrations show a significant preference for lipopeptides over PE and PG lipids. As such, decreased PG–PE interactions could destabilize a bacterial bilayer by making it more susceptible to structural defects and permeation by polar and lipophilic molecules (Tari and Huang 1989).

### Lateral Diffusion

The lateral diffusion coefficient ($D_{\mathrm{L}}$) describes the long-range motion of lipids along the *XY*-bilayer plane. The $D_{\mathrm{L}}$ values obtained are presented in Table 2. For PE and PG lipids, our values of $5.42 \pm 0.25$ and $5.01 \pm 0.25 \times 10^{-7}\, \hbox {cm}^{2}/\hbox {s}$ for the bacterial system are about $\sim 50\%$ higher than the ones previously reported (Hong et al. 2014; Murzyn et al. 2005) for 3:1 PE:PG systems using atomistic simulations. Lipid lateral diffusion has been shown to be highly sensitive to the choice of force fields and simulation conditions (Poger and Mark 2012), and hence we attribute the differences observed to the use of a different force field (Murzyn et al. 2005) and a lower temperature (Hong et al. 2014) in the previous studies. Considering the effect of the addition of lipopeptides, Table 2 shows a drastic reduction by about $\sim\,$85% in lipid lateral diffusion with lipopeptide concentrations at 10 and 25 mol%. At 40 mol%, a similarly substantial (although slightly less pronounced) reduction of $\sim\,$75% can be observed. Overall, it is evident that the presence of cationic lipopeptides markedly reduces the lipid lateral diffusion, even at lower (10%) concentrations. As far as we are aware, no other results have been previously reported in literature on lateral diffusion of bacterial membrane lipids in the presence of cationic lipopeptides.

### Lateral Pressure Profile

The lateral pressure profile ($\varPi (z)$) describes the non-uniform, depth-dependent distribution of lateral stresses across the bilayer normal. An assessment of $\varPi (z)$ can provide molecular-level insights into the interactions between lipids and other membrane constituents (such as proteins/peptides) and an estimate of the overall mechanical stability of the bilayer. Figure 7 depicts $\varPi (z)$ for all the simulated bilayer systems. The pure bacterial bilayer displays a trend which is characteristic of pure phospholipid bilayers in general (Cantor 1999; Orsi and Essex 2011; Ding et al. 2015). In particular, a large positive pressure peak of $\sim\,$340 bar can be seen at the interface of the *water* and *heads* region at about $\sim\,$2.7 nm from the bilayer center. Such positive pressures indicate net repulsive forces as a result of steric and electrostatic interactions between the water molecules and lipid headgroups. Upon proceeding deeper into the bilayer, the pressure dips to a minimum at $\sim\,$1.8 nm forming sharp negative pressure troughs at the interface between the *heads* and *tails* region. Such negative pressures indicate net attractive forces of hydrophobic nature (Marsh 2007). The pressure profile in the bilayer center (*tails* region) is primarily characterized by three positive pressure peaks, that are postulated to arise from loss of entropy due to tight packing of lipid chains in the bilayer core (Mukhin and Baoukina 2005; Orsi et al. 2008).

Upon addition of lipopeptides, significant changes in $\varPi (z)$ can be noted. Regarding the positive pressure peak at the interface of *water* and *heads* region, the magnitude decreases substantially for all systems, while the peak width increases remarkably for bilayers at 25 and 40% lipopeptide concentrations. Deeper in to the bilayer at the heads–tails interface, the large hydrophobic troughs are only slightly affected at 10% lipopeptide concentration (increase of $\sim\,$3.7% in attractive pressures), but undergo a noticeable change at 25% and a substantial one at 40%; a major shift of the pressure trough occurs towards the bilayer core along with a decrease in pressure magnitudes of $\sim\,$150 bar and $\sim\,$205 bar at the two concentrations, respectively. Thus, with respect to the *tails* region, a gradual increase in lipopeptide concentration brings about a notable decrease in $\varPi (z)$, an effect which can be correlated with the decrease in order parameters noted earlier.

In general, it is apparent that the lateral pressure profiles for bacterial bilayer are affected in the presence of AMLPs. The changes are most pronounced for the 40% AMLP system, an effect analogous to that observed previously for other properties. Additional quantitative analysis of the lateral pressure profiles based on elastic parameters is reported in the following section.

### Elastic Properties

Fundamental elastic properties of lipid bilayers can be analytically related to the lateral pressure profile $\varPi (z)$, with the product of monolayer bending rigidity modulus ($\kappa ^{m}$) and monolayer spontaneous curvature ($\text {c}^m_0$) being equal to the first integral moment of $\varPi (z)$:

3 $$\begin{aligned} \kappa ^{m}\text {c}^m_0 = \int ^l_0 z\varPi (z)dz \end{aligned},$$

where $z=l$ is in the water phase and $z=0$ is the bilayer core (Szleifer et al. 1990; Lipowsky and Sackmann 1995; Orsi and Essex 2013). With a standard numerical integration using the trapezoidal rule, the first integral moment of $\varPi (z)$ was obtained. Subsequently, $\kappa ^{m}$ was evaluated using the empirical relation (Rawicz et al. 2000) $\kappa ^{m}=k_{\mathrm{A}}(d_{\mathrm{HH}} - 10)^{2}/48$, with $d_{\mathrm{HH}}$ the bilayer thickness and $k_{\mathrm{A}}$ the area compressibility modulus (values reported in the supplementary information, Table S1). The spontaneous curvature $\text {c}^m_0$ was then simply obtained as the ratio between the first integral moment of $\varPi (z)$ and the monolayer bending modulus $\kappa ^{m}$. The values of the elastic properties for all simulated bilayers are reported in Table 3.

The bending rigidity modulus $\kappa ^{m}$ is arguably the single most important quantity in membrane biophysics (Watson et al. 2012) and determines the membrane’s ability to resist bending in a number of biologically relevant processes such as membrane fusion (Liu et al. 2006), membrane trafficking of molecules (McMahon and Gallop 2005), and endocytosis (Chernomordik et al. 1995). As such, larger values of $\kappa ^{m}$ correspond to greater rigidity of the bilayers. Our value of $\kappa ^{m}$ for the 2:1 PE:PG bacterial bilayer is consistent with a previous one measured using a coarse-grained (CG) 3:1 PE:PG model (Fowler et al. 2016). Regarding the effect of lipopeptides, our results show an 11% increase in $\kappa ^{m}$ with addition of 10% lipopeptide concentration. Interestingly, a further increase in lipopeptide concentration to 25% leads to a decrease of $\kappa ^{m}$ down to $4.32\cdot 10^{-20}$ J, a value close to that of the pure bacterial bilayer. In the system with 40% lipopeptide concentration, $\kappa ^{m}$ drops sharply to $1.55\cdot 10^{-20}$ J, a decrease of up to $\sim$65% as compared to the bacterial bilayer. Such a major drop can be correlated with the previously observed substantial decrease in magnitudes of the pressure profiles for the 40% AMLP system, as a reduction in internal stresses is expected to induce lower membrane rigidity.

In symmetrical bilayers, the monolayer spontaneous curvature $\text {c}^m_0$ determines the intrinsic tendency of monolayers to curl if allowed to bend freely, rather than being constrained to a flat configuration as part of a lamellar bilayer structure (Lewis and Cafiso 1999). A positive $\text {c}^m_0$ indicates a monolayer’s tendency to bend towards the hydrocarbon chains and away from the aqueous phase (tendency to form micelles), while a negative $\text {c}^m_0$ indicates the opposite tendency of bending towards the water phase (tendency to form inverse phases). In general, the more $\text {c}^m_0$ deviates from 0 in a flat system, the more elastic energy is stored in the monolayer; beyond a certain threshold (depending on the system), increases in $\text {c}^m_0$ may destabilize the flat structure. A small magnitude for $\text {c}^m_0$ (close to zero), of either sign, indicates no significant tendency to curl, corresponding to a stable flat lamellar phase. In Table 3, it can be noted that $\text {c}^m_0$ values for all systems are characterized by very low magnitudes, indicating high stability of the flat lamellar bilayer phases. As far as we know, this is the first study either in experiments or simulations to report $\text {c}^m_0$ values for a model bacterial bilayer incorporating lipopeptides. Overall, our results show that the spontaneous curvature for a 2:1 PE:PG bacterial bilayer is not significantly sensitive to the presence of the C16-KKK lipopeptides in it.

### Dipole Potential

The dipole potential profiles ($\varPsi (z)$) for all simulated systems are shown in Fig. 8. Profile decompositions highlighting the individual contributions of ions, water, and lipid/lipopeptide molecules can be found in the supplementary information (Fig. S7). As shown in Fig. 8, all profiles share similar qualitative features, regardless of the different lipopeptide concentrations. However, there are important concentration-dependent differences in the magnitudes of the potentials, whereby increasing amounts of C16-KKK induce increasingly larger values of $\varPsi (z)$ across the $water-heads$ interface. Beginning from a value of 0 V in the water phase, the potential rises rapidly across the lipid *heads* region forming local peaks at around $\sim\,$1.4 nm from the bilayer core, with values ranging from $\sim\,$0.5 V for the pure bacterial system to $\sim\,$0.6 V for the systems with 25 and 40% lipopeptides, with the lower 10% concentration displaying values in-between. The profiles then dip, forming local minima at $\sim\,$0.8 nm from the bilayer center, which roughly corresponds to the double bonds in the palmitoyl chains of lipids. In the membrane core, the dipole potential forms a global maximum for each of the individual profiles, with values of $\sim\,$0.71 V, $\sim\,$0.74 V, and $\sim\,$0.78 V for the bacterial, 10, and 25% AMLP systems, respectively. Interestingly, the 40% AMLP system breaks the trend and forms the lowest global maximum at $\sim\,$0.68 V. This can be related to the order parameters, which we observed to drop sharply for the 40% system; increased disorder in the *tails* region reduces dipolar alignment and ultimately leads to a decreased potential magnitude. As far as we are aware, there are no previous reports in literature on the dipole potential for bacterial lipid bilayers.

### Discussion and Conclusion

In recent times, there has been growing interest in developing short cationic antimicrobial lipopeptides (AMLPs) as a class of compounds that possess antimicrobial properties with fewer drawbacks compared to standard antimicrobial peptides (AMPs). While previous experimental studies have focused on quantifying bactericidal activities of various lipopeptides (Makovitzki et al. 2008, 2007, 2006), previous computational research focused on their self-assembly process and binding interactions with model bacterial bilayers (Horn et al. 2013; Sikorska et al. (2014). In this work, we studied mixed bilayer systems where the cationic C16-KKK AMLPs are fully incorporated into a PE/PG bacterial bilayer. In particular, we deploy atomistic MD simulations to investigate the effect of C16-KKK AMLPs on a number of key physical properties of a model 2:1 PE:PG bacterial bilayer system, at concentrations of 10, 25, and 40 mol%. A concise summary of all conclusions is reported in Table 4. With the exception of the monolayer spontaneous curvature, the properties investigated were shown to be notably affected by the cationic AMLPs, especially at the higher 25 and 40 mol% concentration levels.

In particular, at 10% AMLP concentration, the lipopeptides appear to have a condensing effect on bacterial bilayers, characterized by a significant decrease in the area per lipid. The AMLPs acyl chains are fully inserted in the bilayer core, while their lysines are largely hydrated and interact with both PG and PE headgroups. There is an overall increase in bilayer order, accompanied by a marked reduction in lateral diffusion for both lipids. While PG lipids preferentially interact with PEs in bacterial bilayers, in the 10% AMLP system anionic PGs intuitively show preference for interaction with cationic lipopeptides while like-charge repulsions keep PG–PG and AMLP–AMLP interactions to a minimum. Increases in lipopeptide concentrations to 25 and 40% were observed to bring about significant disruption to the bacterial lipid bilayer. The main effects involve an increase in lipid chain interdigitation, a decrease in order parameters and bending rigidity modulus, and an enhanced degree of spontaneous permeation of water molecules in the bilayer core, especially in the 40% AMLP system. Thus, membrane stability is particularly impaired at the highest concentration of 40%, which corresponds to the ratio 1 AMLP : 1 PE : 2 PG.

The lateral pressure profile is also substantially altered at 40% AMLP concentration, as we observed a systematic and substantial reduction in pressure magnitudes throughout the bilayer. Quantitatively, the pressure differences between the bacterial and 40% AMLP systems are on the order of hundreds of bars, and changes of this size may play a role in membrane protein functionality (Cantor 1997). For example, the bacterial mechanosensitive channel (MS) is thought to play an important role in regulating turgor pressure around the cell (Perozo and Rees 2003), and the gating of these channels found in inner bacterial membranes of *Escherichia coli* is shown to be affected by changes in lateral pressure (Gullingsrud and Schulten 2004; Moe and Blount (2005). Previous studies have shown that, in response to a rapid osmotic shock, many of the cytoplasmic components such as solutes/water are ejected in to the surroundings by opening of the MS channels to relieve the osmotic imbalance (Perozo and Rees 2003). Thus a permanent leakage through the channel can lead to the death of bacteria, and an understanding of the relevant gating mechanism could lead to the development of new or improved antimicrobial agents. Our results also suggest an antimicrobial mechanism of action whereby cationic lipopeptides cause increased rates of water permeation events in the bilayer core, as quantified by the increase in water permeation coefficients, especially for the 40 mol% AMLP system. We speculate that the biological effects of AMLPs mainly arise from locally disordering the bilayer core and inducing leakage in inner bacterial membranes.

Overall, our results provide a quantitative molecular-level understanding of the experimental observations that cationic lipopeptides in general, and the C16-KKK AMLP in particular, are highly potent towards bacterial cells and operate by means of destabilizing their membranes (Makovitzki et al. 2008, 2007, 2006). Our work provides the basis for future simulations of more complex bacterial membranes, especially including mechanosensitive proteins, aimed at quantifying potential AMLP effects on protein conformational states.

## Electronic supplementary material

Below is the link to the electronic supplementary material.

(PDF 6656 kb)
