# Supplementary material for: Interaction of Antimicrobial Lipopeptides with Bacterial Lipid Bilayers
Source: J Membr Biol. 2019 May 16;252(4):317–29. doi: 10.1007/s00232-019-00068-3 (PMC6790193; doi:10.1007/s00232-019-00068-3)
Supplement: Supplementary file 1 — (PDF 6656 kb) [file 232_2019_68_MOESM1_ESM.pdf]

Supporting Information

Interaction of antimicrobial lipopeptides with bacterial lipid bilayers

Ganesh Shahane,[†] Wei Ding,[‡] Michail Palaiokostas,[‡] Helena S. Azevedo,[‡] and Mario Orsi[*]

[†]*Institute of Bioengineering, Queen Mary University of London*
Mile End Road, London E1 4NS, UK

[‡]*School of Engineering & Materials Science, Queen Mary University of London*
Mile End Road, London E1 4NS, UK

[*]*Department of Applied Sciences, University of the West of England*
Coldharbour Lane, Bristol BS16 1QY, UK

E-mail: Mario.Orsi@uwe.ac.uk

Numerical values

Table S1: Numerical values of properties of interest.

System	POPE-A_L (Å^2)	POPG-A_L (Å^2)	d_{HH} (Å)	k_A (dyn/cm)	P_W (μm/s)
Bacterial	56.6 ± 0.2	63.1 ± 0.2	41.3 ± 0.4	219.5 ± 14.0	19.8
10% AMLP	54.8 ± 0.1	60.9 ± 0.4	41.6 ± 0.2	239.5 ± 20.4	15
25% AMLP	55.1 ± 0.3	61.5 ± 0.2	39.4 ± 0.2	240.6 ± 28.8	24.6
40% AMLP	59.4 ± 0.6	62.1 ± 0.6	34.1 ± 0.13	128.2 ± 4.6	54

Electron density profiles

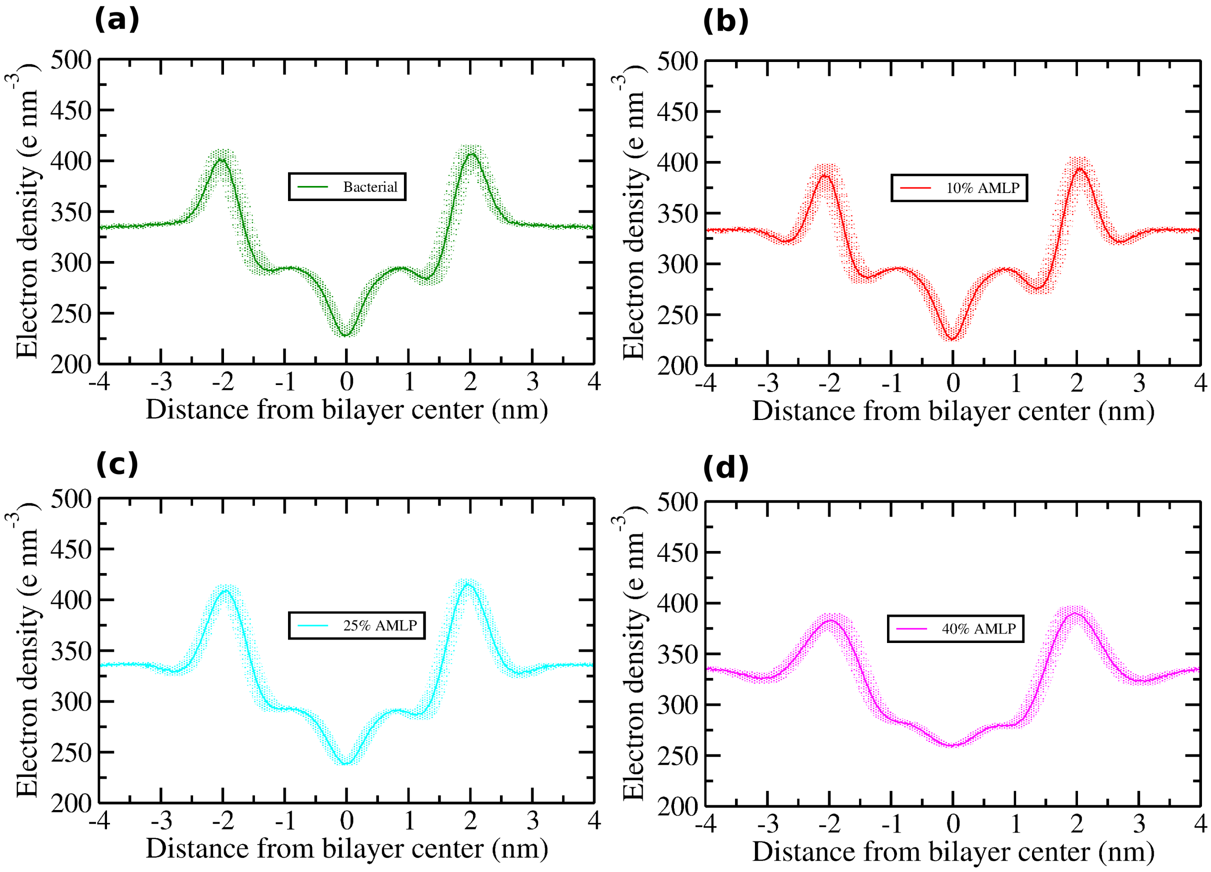

Figure S1: Electron density profiles with error bars for (a) bacterial, (b) 10% AMLP, (c) 25% AMLP and (d) 40% AMLP systems.

Phosphate electron density profiles

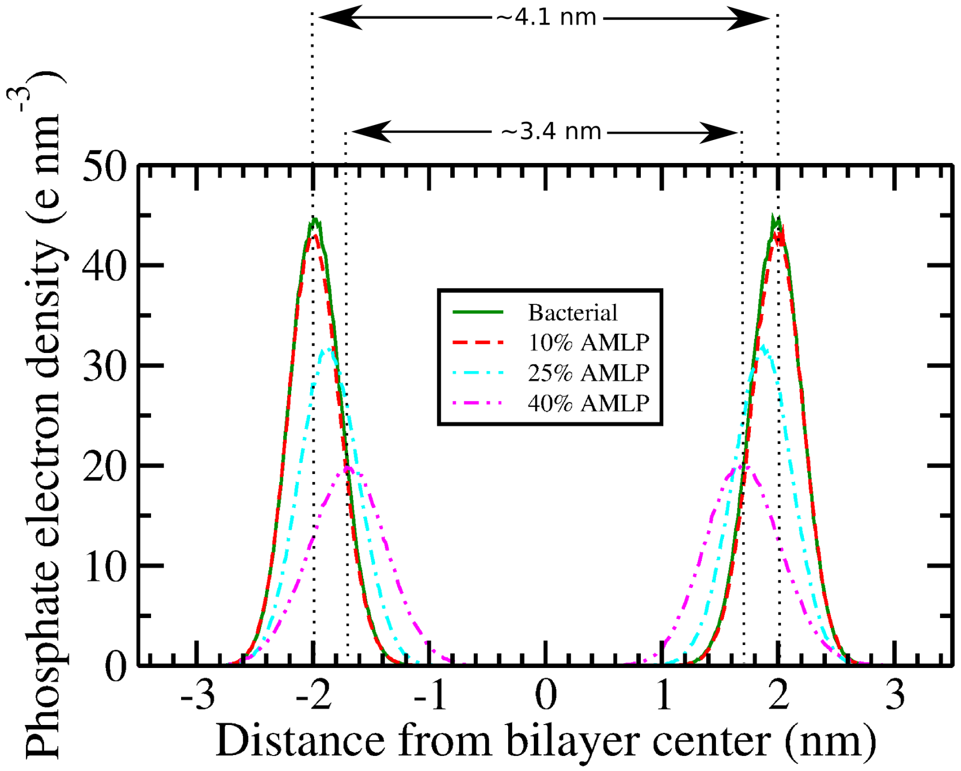

Figure S2: Electron density profiles for phosphate atoms in all four simulated systems. The peaks here better highlight the differences in their bilayer thicknesses.

Interdigitation of lipid chains

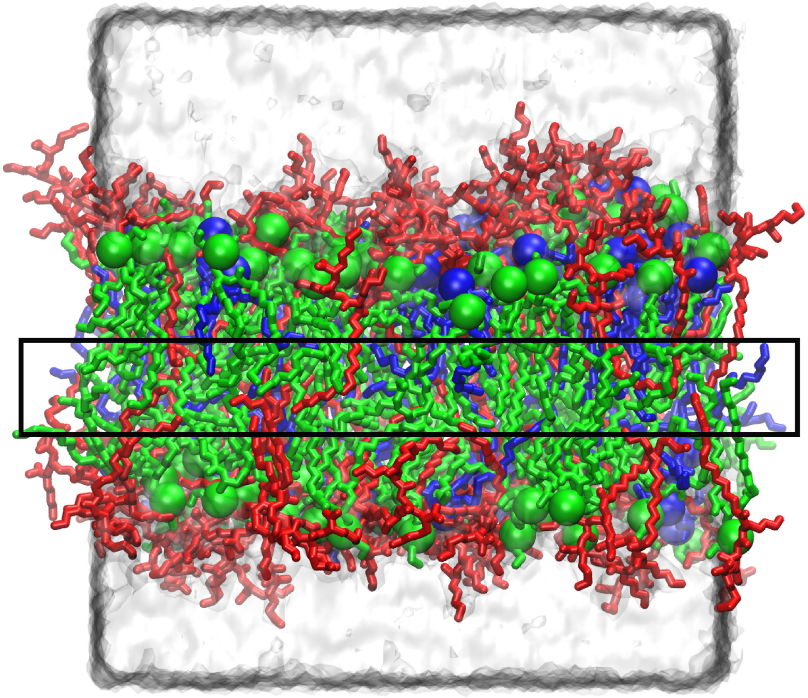

Figure S3: Lipid chain interdigitation as seen in the bilayer core of the 40% AMLP system. The PEs are coloured green, PGs are blue and AMLPs are red.

Water permeation

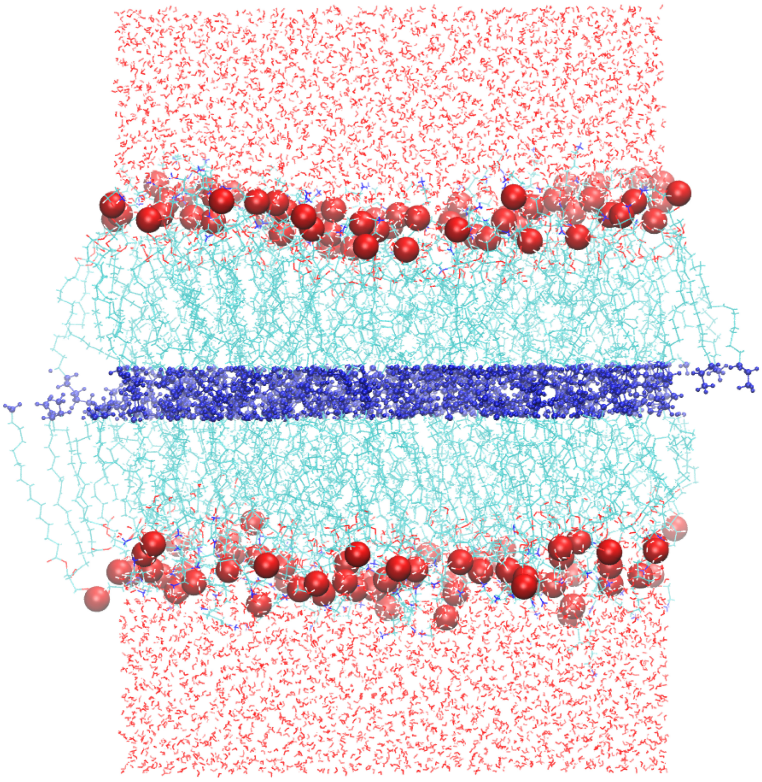

Figure S4: Central region of bilayer core defined for water permeation calculations as shown using the CPK representation and coloured in dark blue.

Lateral pressure profiles

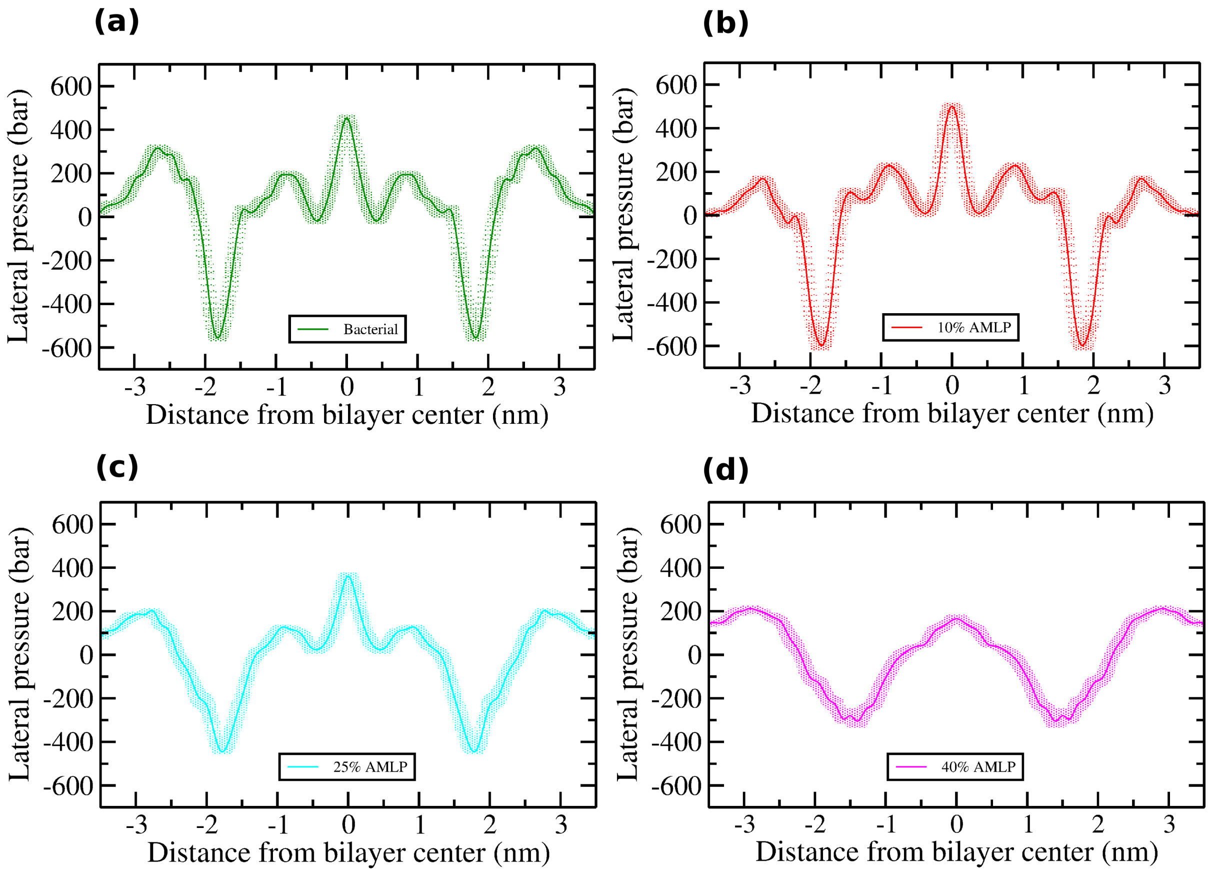

Figure S5: Lateral pressure profiles with error bars for (a) bacterial, (b) 10% AMLP, (c) 25% AMLP and (d) 40% AMLP systems.

Dipole potential profiles

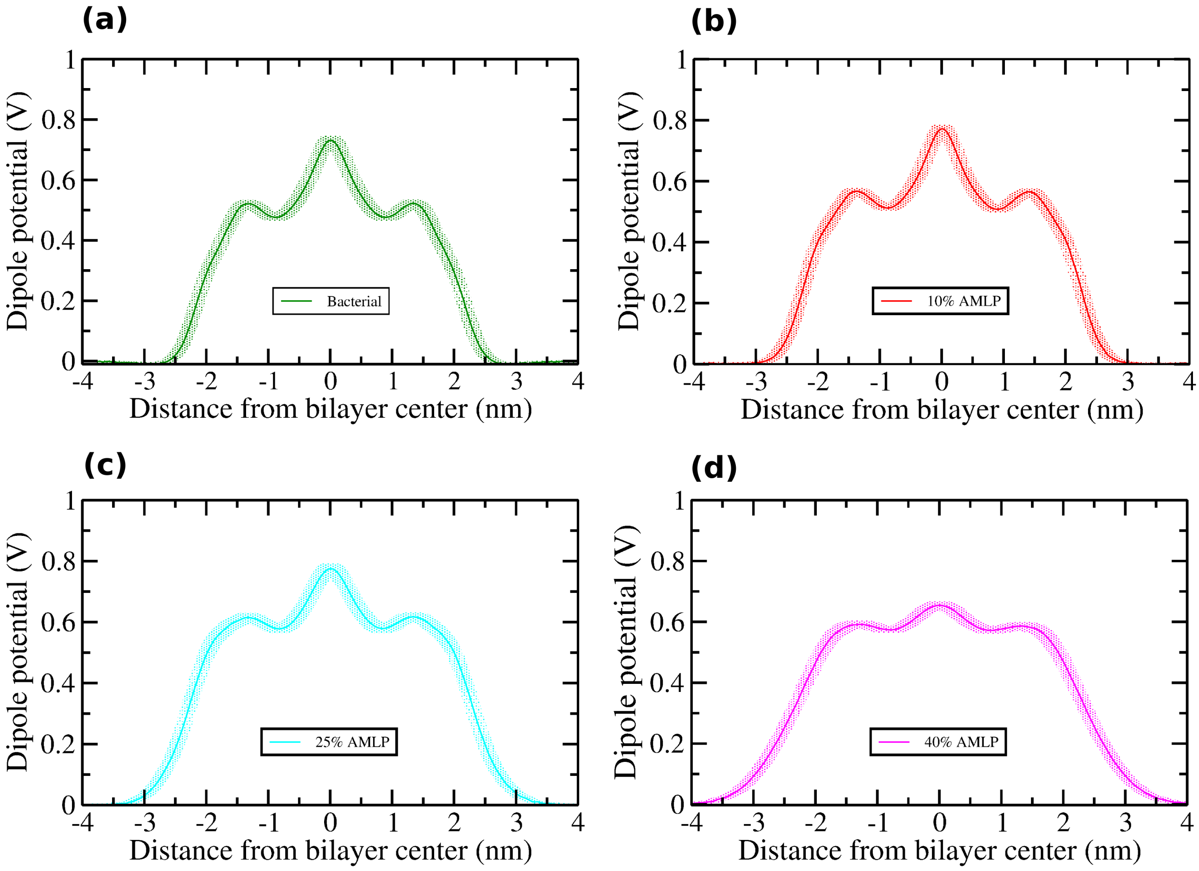

Figure S6: Dipole potential profiles with error bars for (a) bacterial, (b) 10% AMLP, (c) 25% AMLP and (d) 40% AMLP systems.

Dipole potential contributions

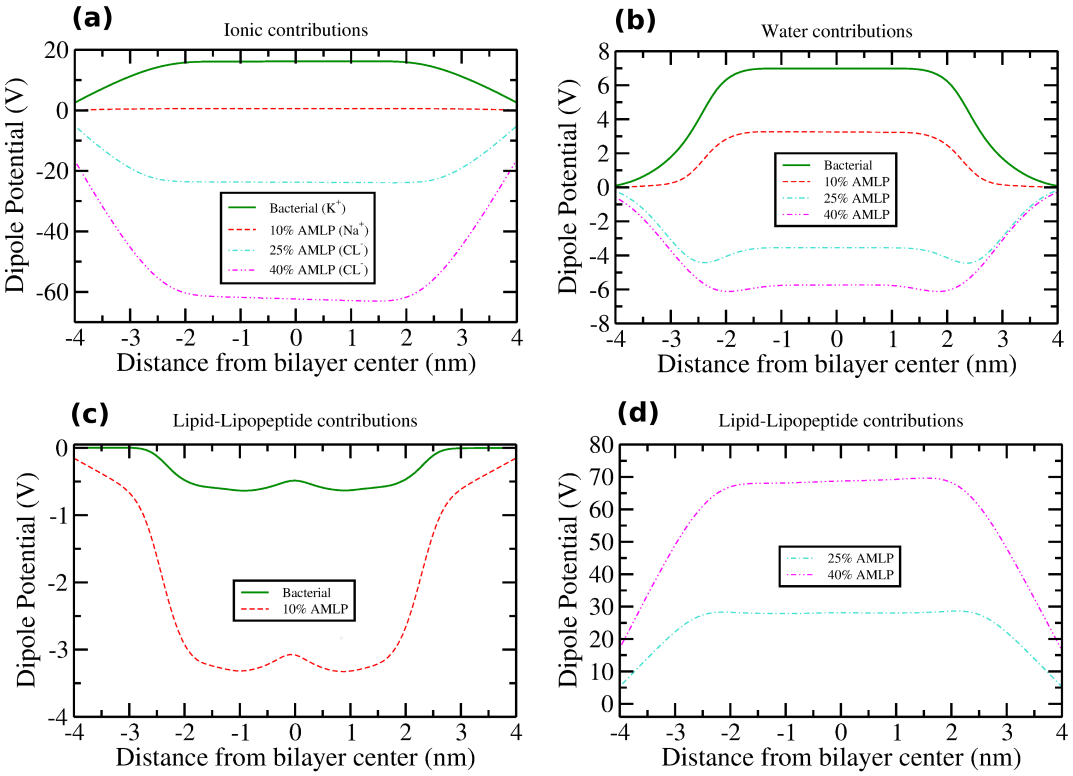

Figure S7: Individual contributions of the dipole potential profile from (a) ions, (b) water and (c-d) lipids-lipopeptides. For all systems, the net positive dipole potential comes from a sum total of larger positive and negative contributions, which come from different components in different systems. For the bacterial and ”10% AMLP” systems the net positive contributions come from water and ions while the negative ones come from lipid and lipopeptides. On the other hand, the positive contributions come from lipids and lipopeptides for the 25 and 40 mol% AMLP systems, while the negative ones come from water and ions.
